# Clinical and microbiological diagnosis of oral candidiasis

**DOI:** 10.4317/jced.51242

**Published:** 2013-12-01

**Authors:** Laura Coronado-Castellote, Yolanda Jiménez-Soriano

**Affiliations:** 1 Dentistry. Master of Oral Medicine and Oral Surgery. Faculty of Medicine and Dentistry. University of Valencia. Spain; 2 Associate Professor of the Department of Stomatology. Faculty of Medicine and Dentistry. University of Valencia. Spain

## Abstract

Introduction: Candidiasis or oral candidiasis is the most frequent mucocutaneous mycosis of the oral cavity. It is produced by the genus Candida, which is found in the oral cavity of 53% of the general population as a common commensal organism. One hundred and fifty species have been isolated in the oral cavity, and 80% of the isolates correspond to Candida albicans, which can colonize the oral cavity alone or in combination with other species. Transformation from commensal organism to pathogen depends on the intervention of different predisposing factors that modify the microenvironment of the oral cavity and favor the appearance of opportunistic infection. The present study offers a literature review on the diagnosis of oral candidiasis, with the purpose of establishing when complementary microbiological techniques for the diagnosis of oral candidiasis should be used, and which techniques are most commonly employed in routine clinical practice in order to establish a definitive diagnosis. Materials and methods: A Medline-PubMed, Scopus and Cochrane search was made covering the last 10 years. Results: The diagnosis of oral candidiasis is fundamentally clinical. Microbiological techniques are used when the clinical diagnosis needs to be confirmed, for establishing a differential diagnosis with other diseases, and in cases characterized by resistance to antifungal drugs. Biopsies in turn are indicated in patients with hyperplastic candidiasis. Staining (10% KOH) and culture (Sabouraud dextrose agar) are the methods most commonly used for diagnosing primary candidiasis. Identification of the individual species of Candida is usually carried out with CHROMagar Candida®. For the diagnosis of invasive candidiasis, and in cases requiring differentiation between C. albicans and C. dubliniensis, use is made of immunological and genetic techniques such as ELISA and PCR.

** Key words:**Clinical, oral candidiasis, microbiology.

## Introduction

Mycoses comprise a series of infectious diseases caused by the pathogenic action of fungi. Although most fungal species are present as saprophytes in the environment and in plants, where they play a key role in the natural recycling of organic material, some of them are able to cause disease in humans. Such conditions manifest as allergic processes caused by the inhalation of spores, intoxication as a result of the ingestion of harmful substances (mycetismus or mushroom poisoning and mycotoxicosis), or infections, since some fungal species are able to colonize, invade and multiply in different body tissues and organs, causing mycosis in a previously healthy host (primary pathogenic fungi) or in individuals with diminished defense mechanisms (opportunistic fungi). The clinical profile of mycosis varies greatly depending on the causal agent, the location of the infection, and the predisposing factors of the patient. One of the most widely used practical classifications of mycoses is based on the location of the infection. In this sense, mycoses can be classified as superficial, cutaneous or mucocutaneous, subcutaneous, and deep or systemic. Mucocutaneous mycoses affect skin and mucosal membrane integrity, and moreover produce inflammation.

Candidiasis or oral candidiasis, produced by fungi of the genus *Candida* (*Candida spp.*), is the most frequent mucocutaneous mycosis of the oral cavity. *Candida* is found in the oral cavity of 53% of the general population as a common commensal organism. One hundred and fifty species of this genus have been isolated in the oral cavity, and 80% of the isolates correspond to *Candida albicans*, which can colonize the cavity alone or in combination *Candida glabrata* or *Candida tropicalis* (observed in 7% of all healthy people and in 80% of all patients with candidiasis). Recently, *Candida dubliniensis* has been isolated in patients with human immunodeficiency virus (HIV) infection. This species is important, since it is associated with very severe candidiasic mucositis of the oral cavity, esophagus and other locations, and is moreover resistant to antifungal treatment. Despite the above observations, the prevalence of oral candidiasis is not well known, since in many cases these processes go undetected by the clinician. Nevertheless, the diagnosis of the disease is very important, since it may be the first manifestation of a systemic disorder, including possible HIV infection (1).

The pathogenesis of candidiasis combines three factors: host, fungus and oral microenvironment-modifying factors. The host predisposing factors include endocrine alterations (diabetes mellitus, pregnancy, renal failure and hyperthyroidism)(2,3), immune depression (normally associated to antineoplastic treatments or immunosuppression in transplant patients, as well as agammaglobulinemia or cellular immune defects)(1,4-8), acquired immunodeficiency syndrome (AIDS) or hematological and immune disorders such as agranulocytosis (neutropenia). Other predisposing conditions are malignant diseases such as lymphomas or leukemias, aplastic anemia, drug treatments (long-term administration of broad spectrum antibiotics, corticosteroids, antidepressants, antineoplastic drugs and immunosuppressants)(9-11), hyposialia (produced by disorders such as Sjögren’s disease, drugs or radiotherapy), and terminal or end-stage systemic diseases (10,12,13). The oral microenviron-ment-modifying factors in turn include poorly fitting dentures (14-17), loss of vertical dimension, chronic antiseptic use, prolonged dummy use in children, poor oral hygiene (16), smoking and alcoholism (10,18). The genus *Candida* is the most common of the many types of fungi found in the oral cavity. The species of *Candida spp*. isolated in the oral cavity are *C. albicans, C. tropicalis, C. parapsilosis, C. krusei, C. guillermondii, C. glabrata and C. dubliniensis. Candida albicans* possesses pathogenicity factors that allow it to develop disease more frequently than other species of *Candida*. The formation of germinal tubes and the presence of certain glycoproteins such as mannose and glucose in the fungal wall facilitate adherence to cell membranes and receptors. At the same time, the presence of germinal tubes and the production of phospholipase C also facilitate fungal invasion. Some components of these yeasts alter the host defense mechanisms (e.g., inhibiting phagocytosis). On the other hand, certain polysaccharides in the fungal wall induce suppressor T lymphocytes, and mannan found in the wall can interfere with antigen presentation, inhibiting the immune defense response. Tissue damage results from direct action of the microorganism but also from the host defenses developed against tissue invasion, including IgE-mediated immune allergic reactions against the fungal antigens and delayed hypersensitivity reactions – as in chronic mucocutaneous candidiasis. Thus, in order for *Candida* to produce infection, it must colonize, invade and multiply.

*Candida* is a rounded and oval-shaped yeast measuring 3-30 µm in diameter. It reproduces asexually through a budding process in which protoplasmic protrusions or buds (blastoconidia) emerge from the mother cell and grow until they finally detach to form a new cell. The daughter cells occasionally do not detach and form chains of cells called pseudohyphae, which can be mistaken for hyphae. The latter are composed of a row of elongated cells enveloped by a cell wall; they globally conform the mycelium (septate and ramified hyphae). In solid culture media, the yeast grows, giving rise to compact colonies that are macroscopically visible after 24-48 hours of incubation. *Candida* must be in the saprophytic phase in order to produce clinical lesions, though over time nutritional and environmental variations modulate its conversion to the mycelial or invasive form. In this phase the yeast keeps its previous virulence intact, and is able to evade macrophage phagocytic action (19,26).

The diagnosis of any of the forms of oral candidiasis is essentially clinical and is based on recognition of the lesions, which can be confirmed by the microscopic identification of *Candida* in the oral samples and/or isolation in culture, among other diagnostic methods. In the case of *Candida*, detection of the fungus in the oral cavity is not indicative of infection, since it is a common commensal organism in this location. A definitive diagnosis of candidiasis requires the confirmation of tissue invasion by *Candida*. For this reason a negative culture result is of greater use in discarding candidiasic infection than a positive culture result in confirming infection. It may be stated that in the absence of clinical manifestations compatible with oral candidiasis, a positive culture result for *Candida* does not mean that the patient has oral candidiasis. The importance of the clinical diagnosis of the disease therefore must be underscored (2).

## Objectives

The present study describes the correct clinical diagnosis of oral candidiasis, the situations in which complementary microbiological techniques for the diagnosis of oral candidiasis should be used, and the techniques most commonly employed in routine clinical practice to establish a definitive diagnosis.

## Material and Methods

A literature search was made using the following key words: “clinical”, “microbiology” and “oral candidiasis”. The key words were validated by the MeSH (Medical Subject Headings) dictionary, with use of the boolean operator “AND” to relate them.

The following limits for inclusion of the studies were established: publications in the last 10 years (2002 2012), publications in English and Spanish, publications of studies in humans, and all articles with level I and II evidence (systematic reviews and clinical trials).

The search for articles was made with the Medline-PubMed, Cochrane and Scopus databases, in that order.

Following application of the limits, two searches were made in each of the databases, using the following key words in English: “Clinical diagnosis AND oral candidiasis” and “Microbiology AND oral candidiasis”. A total of 183 articles were identified, of which 89 were selected after reading the abstracts. Following analysis of the 89 articles, we finally included a total of 37, since those publications that did not fit the aims of the present study were excluded. Of the mentioned 37 articles, 28 were clinical trials and 9 systematic reviews.

## Results and discussion

In the early twentieth century it was seen that oral candidiasis could have different clinical and histopathological manifestations. Posteriorly, a distinction was made between primary oral candidiasis and secondary forms of the disease, in which oral candidiasis merely constituted one of the manifestations of mucocutaneous-systemic candidiasic infection. At present, the most widely used classification is that developed by Holmtup and Axel (2), which contemplates the following presentations: pseudomembranous candidiasis (acute-chronic), erythematous candidiasis (acute-chronic), hyperplastic candidiasis, and associated lesions (prosthetic stomatitis, angle cheilitis, rhomboid glossitis).

Pseudomembranous candidiasis is usually observed in immune depressed individuals, irradiated patients, nursing infants (due to the low pH in the oral cavity and the lack of a stable microbial ecosystem capable of inhibiting development of the fungus), elderly people, and patients with xerostomia or diabetes mellitus. It tends to manifest in the form of whitish-yellow plaques (masses of hyphae, yeasts, cell detritus and desquamated epithelial cells) of a soft and gelatinous consistency that exhibit a centrifugal growth pattern. The lesions appear especially in the oropharyngeal region, cheek mucosa and lateral surfaces of the tongue. The whitish-yellow plaques detach upon rasping, leaving an erythematous zone. Symptoms are scarce (burning and itching sensation). Dysphagia may be observed when the lesions affect the oropharynx (21,24).

Acute erythematous candidiasis is the most common presentation in both immune depressed and immunocompetent individuals, and tends to manifest as a complication of broad spectrum antibiotic treatment. It is characterized by erythematous and atrophic regions located anywhere within the oral cavity, but particularly on the palate and tongue. The filiform papillae disappear, and the dorsal surface of the tongue appears smooth. The condition is usually asymptomatic or is accompanied by mild burning and itching sensation (12,18,22-24).

Chronic hyperplastic candidiasis can manifest in nodular form or as whitish plaques that cannot be attributed to any other disorder, do not detach upon rasping, and are typically located on the cheek mucosa and tongue, and especially bilaterally at both lip retro-commissures. In this form of the disease the *Candida* hyphae are not only found at epithelial surface level but also invade deeper levels where epithelial dysplasia can be observed – with the associated risk of malignization (18,25).

Prosthetic stomatitis manifests as erythematous zones closely related to the base of poorly fitting and very old removable dentures with poor hygiene that produce continuous trauma (14-22). A punctate (Newton 1) or extensive smooth reddened appearance can be observed (Newton 2), or extensive reddening with hyperplastic growth can be noted (Newton 3)(2,18,26). The underlying etiology is multifactorial (dentures, hygiene and microbiological, dietetic and systemic factors), and infection due to Candida can be involved.

Angle cheilitis usually appears in patients with vitamin and iron deficiencies, or staphylococcal infections, as well as in totally or partially edentulous individuals with vertical dimension loss. The consequence is the formation of fissures or cracks at the lip commissures, with the accumulation of saliva that favors the generation of a humid environment and colonization by *Candida*. Over time, the fissures and erosions can produce crusts and pain. These patients typically present mixed infections, with the presence of bacteria such as Staphylococcus aureus or certain streptococci (10,18,27).

Median rhomboid glossitis (MRG) was first described by Brocq in 1914, and affects less than 1% of the general population. It is more commonly seen in males, smokers and diabetic patients. Clinically, MRG affects the posterior region of the back of the tongue, on the midline anterior to the lingual “V”, and rarely the paramedial zone. The condition manifests as an asymptomatic, rounded or rhomboid plaque of firm consistency and an intense red or pink color, secondary to atrophy or the absence of filiform papillae, with limits that are clearly differentiated from the rest of the tongue. There are two distinct clinical presentations: an atrophic or macular and non-elevated (flat) form, or a mamillated, hyperplastic or exophytic lesion that can appear elevated (up to 2-5 mm). In some cases a fissured or lobulate lesion can be observed (10,18,28).

The diagnosis of any of the forms of oral candidiasis is essentially clinical and is based on recognition of the lesions, which can be confirmed by the microscopic identification of *Candida* in the oral samples and/or isolation in culture, among other diagnostic methods. In the case of *Candida*, detection of the fungus in the oral cavity is not indicative of infection, since it is a common commensal organism in this location. A definitive diagnosis of candidiasis requires the confirmation of tissue invasion by *Candida*. This underscores the importance of the clinical diagnosis of the disease (27).

A microbiological study is usually required when there are diagnostic doubts, resistances to antifungal drugs, or when the antifungal drug dose requires adjustment, as in immune depressed patients. Microbiological techniques are also typically used when it is necessary to control the disease with a view to avoiding distant spread of the infection, and where the species of *Candida* must be identified in order to establish the most effective treatment. Biopsies are always required in hyperplastic candidiasis in order to discard the existence of epithelial dysplasia (29).

Identification of the yeasts can be made based on four different criteria: morphological and biochemical (for the diagnosis of oral candidiasis), or immunological and genetic (for the diagnosis of invasive candidiasis or the differentiation of species such as *C. albicans and C. dubliniensis*) (30,31).

**Figure 1:**
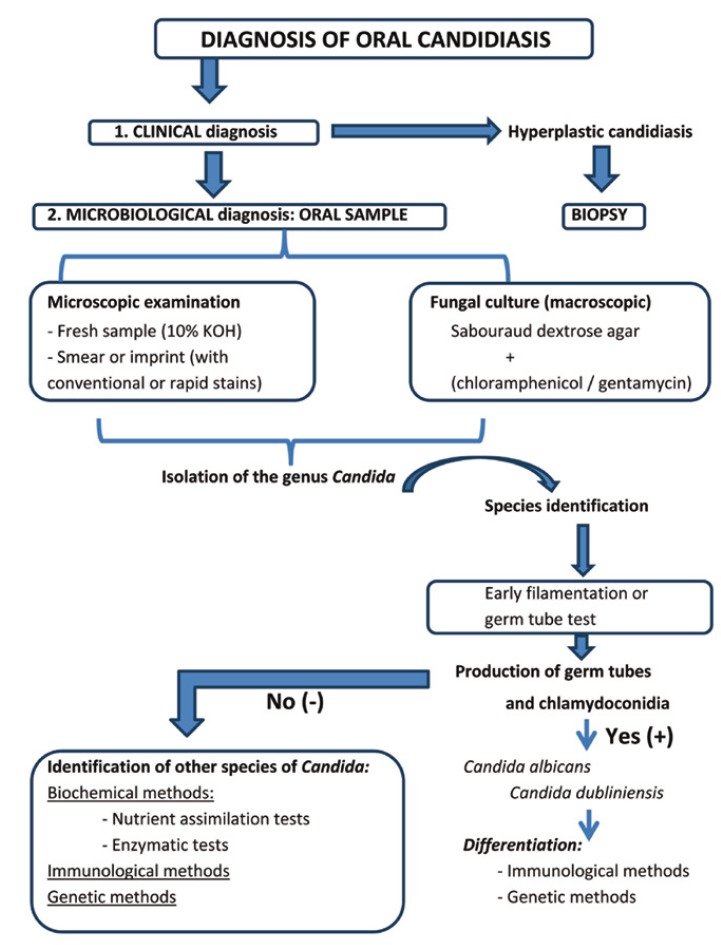
Diagnosis of oral candidiasis.

Although the clinical diagnosis of oral candidiasis is relatively simple, confirmation of the diagnosis can be made by the microscopic observation of *Candida* in oral lesion samples (microscopic morphological criteria) and by isolation in culture (macroscopic morphological criteria) (Fig. 1). Microscopic examination can be made with fresh samples, using 10% potassium hydroxide (KOH), which dissolves the epithelial cells and leaves *Candida* intact, or 15-30% sodium hydroxide (NaOH)(29). It is also possible to prepare smears or imprints of the lesion samples, followed by conventional Giemsa or PAS staining, or rapid techniques such as gram staining. In the case of hyperplastic candidiasis, a biopsy of the lesions is usually obtained and stained with hematoxylin-eosin (showing the yeasts and pseudomycelia of *Candida* with a violet color), PAS or Gomori-Grocott methenamine silver (29-33). Macroscopic observation is made in culture plates. The culture medium used is Sabouraud dextrose agar (SDA), which allows selective fungal growth. Chloramphenicol 0.05 g/l or gentamycin 0.5 g/l is usually added to this medium in order to inhibit bacterial growth. It is also possible to add cycloheximide 0.05 g, since it prevents the overgrowth of other accompanying fungi (Fig. 1). Following incubation (24-48 hours), whitish cottony colonies are observed (7,13,31,33).

Using cultures, we can isolate the genus Candida and identify the species. For the identification of *C. albicans* we use the early filamentation test or germ tube test. Cornstarch agar, Oxgall-Tween-Caffeic acid (TOC), diluted milk and different sera, such as horse serum, can be used for such tests. Horse serum is the most widely used option, with incubation at 37ºC during 2-3 hours (21). Following the incubation period we can observe the characteristic germ tubes of *C. albicans*. The identification of germ tubes and chlamydoconidia is indicative of infection produced by *C. albicans* and/or *C. dubliniensis*, while their absence is indicative of probable infection due to non-*C. albicans* species (Fig. 1).

In this latter case, in which other species of *Candida* must be evaluated, we can use biochemical methods, including enzyme techniques, nutrient assimilation methods and mixed techniques that combine enzymatic and nutrient assimilation tests. The enzyme techniques (chromogenic media, commercial media for the rapid identification of *C. albicans*, and commercial methods for the rapid identification of *C. glabrata*) detect the activity of certain yeast enzymes through the specific hydrolysis of a chromogenic substrate in the presence of an enzyme indicator. Many enzyme techniques are commercially available (CHROMagar Candida®, Candida ID®, Candida ID 2®, CHROMagar-PAL®, Brilliance® Candida Agar, Colorex®, Chromogen albicans®, Albicans ID2®, Candiselect®, Fluoroplate® and Agar SDCA-MUAG®)(5,7,8,14,22). However, the most frequently used system is CHROMagar Candida®, where *C. albicans* grows forming smooth green colonies, *C. tropicalis* forms smooth blue colonies, and *C. krusei* forms rough pink colonies (3,8,9,26). Another widely used chromogenic method is Candida ID®, where *C. albicans* grows to form smooth blue colored colonies, *C. tropicalis* and C. guillermondii generate pink colonies, and the rest of the species appear as white colonies (22) (Fig. 1). Regarding the sensitivity and specificity of the chromogenic methods, CHROMagar Candida® and Candida ID® are simple, easy to use, accessible and offer good sensitivity and specificity, since they allow presumptive identification of most species of Candida (Table  Table 1, Table 2). As can be seen in Table  Table 1 and  Table 2, Sahand et al. (27) propose the use of CHROMagar Candida® with Pal agar (CHROMagar-PAL®), since it allows easy differentiation between *C. albicans and C. dubliniensis* even in mixed cultures (*C. tropicalis, C. krusei and/or C. glabrata*) – reaching a sensitivity of 97% and a specificity of 100%, approximately.

**Table 1:**
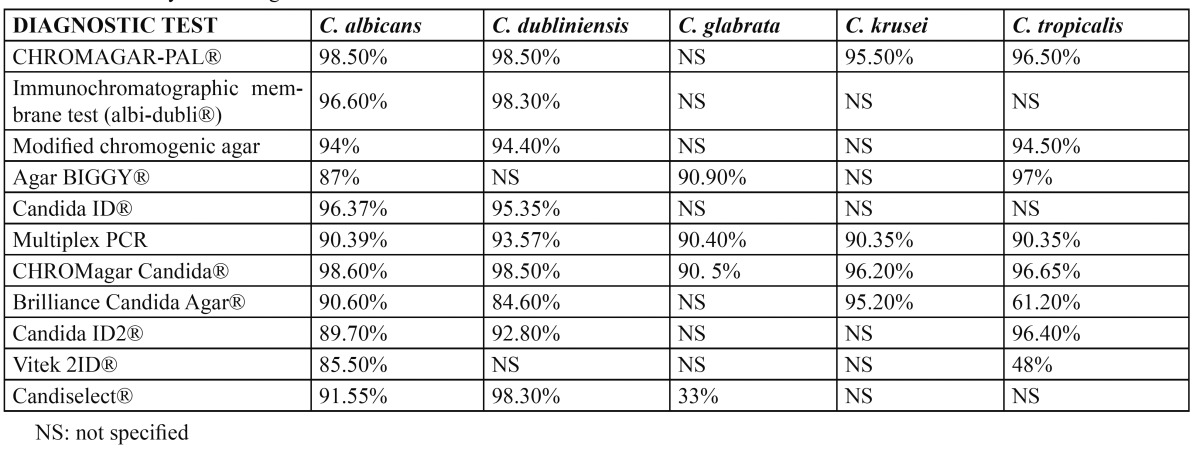
Sensitivity of the diagnostic tests.

**Table 2:**
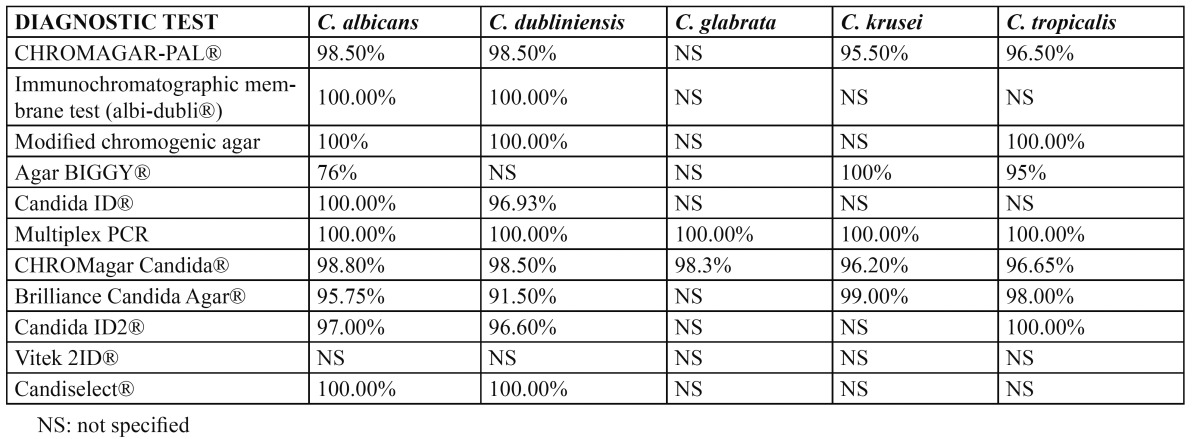
Specificity of the diagnostic tests.

Among the range of biochemical techniques, the nutrient assimilation tests evaluate fungal capacity to use different sugars as sole carbon source. Media are used containing all the elements essential for growth, except a carbon source. A certain sugar is subsequently added to the medium, and the capacity of the fungus to assimilate that sugar is evidenced by its growth in the culture medium. This technique yields what is known as an auxonogram. The latter can be obtained by conventional techniques, using commercial micromethods, or automated systems. Many techniques are currently available and facilitate the use of these tests – making them faster but also more expensive. The nutrient assimilation tests include Auxacolor®, Uni-Yeast-Tek®, API 20 C AUX®, Galeria ID32C® and VITEK 2ID®. Lastly, among the biochemical methods, a number of commercial techniques combine enzymatic tests and nutrient assimilation techniques, such as Rapid Yeast Plus System® and Fongiscreen 4H® (Fig. 1).

The species *C. dubliniensis* produces germ tubes and chlamydoconidia in the same way as *C. albicans*. In order to distinguish between them we therefore need to apply immunological and genetic criteria (Fig. 1). Such criteria are also used for the diagnosis of invasive candidiasis. Immunological criteria are used in the case of patients with complicated clinical conditions (such as immune depressed individuals), when it proves difficult to obtain deep samples, or when there are long waiting intervals between cultures (18,28,31). The immunological techniques include methods based on the detection of mannan / anti-mannan (Platelia™ Candida Ab/Ac/Ak) (32), the detection of antimycelial antibodies (Candida albicans IFA IgG), the detection of other antibodies (Candida Detect™), and the detection of (1-3) β-D-glucan (Fungitec G, Wako, B-Gstar) (25). The immunological procedures likewise include latex particle agglutination tests, based on the use of specific monoclonal antibodies (Bichro-latex albicans®, Krusei-color®, Bichro-Dubli®).

The genetic techniques offer high sensitivity and specificity, and allow identification of the pathogen without having to perform cultures. Furthermore, samples from patients receiving antifungal treatment can be used (19) ( Table 1, Table 2). However, these techniques are more expensive, and are not available in most hospital centers. The molecular diagnosis is based on nucleic acid hybridization and amplification techniques (5,33,36-38). The objective of the genetic systems is to identify yeasts directly in clinical samples without the need to extract nucleic acids – thereby eliminating the culture time and lowering the costs. Bosco-Borgeat et al. (39) and Liguori et al. (40) propose Multiplex polymerase chain reaction (PCR) assays, since they meet the aforementioned objective; however, although these assays are valid, they remain expensive.

## Conclusions

The diagnosis of oral candidiasis is fundamentally clinical. A microbiological diagnosis is performed when the clinical diagnosis requires confirmation; for establishing a differential diagnosis with other diseases; in cases characterized by resistance to antifungal drugs; and in hyperplastic candidiasis, where biopsies are made. The methods most frequently used for the diagnosis of primary candidiasis are smears, stains (10% KOH) and cultures (Sabouraud dextrose agar). In determining the species of *Candida*, the most widely used techniques are CHROMagar Candida® and *Candida* ID®, since they offer good sensitivity and specificity and are accessible and simple to use – allowing the presumptive identification of most *Candida* species. When diagnosing invasive candidiasis, and in cases where a differentiation between *C. albicans and C. dubliniensis* is required, use is made of immunological techniques such as ELISA and genetic methods such as PCR assay. Although a new method has been proposed, known as CHROMagar-PAL®, which combines CHROMagar Candida® and Pal agar and allows simple and easy differentiation between *C. albicans and C. dubliniensis* even in mixed cultures, Multiplex PCR is currently advised since it identifies yeasts directly in clinical samples without the need for nucleic acid extraction - thereby eliminating the culture time and lowering the costs, though the method is still expensive.
